# Amin-modified graphene oxide as a promising adsorbent for selective extraction of dexamethasone adulterant from herbal supplements

**DOI:** 10.55730/1300-0527.3477

**Published:** 2022-05-12

**Authors:** Mahboob NEMATI, Ali ZAYER, Samin HAMIDI

**Affiliations:** 1 Food and Drug Safety Research Center, Tabriz University of Medical Sciences, Tabriz, Iran; 2 Department of Pharmaceutical and Food Control, Faculty of Pharmacy, Tabriz University of Medical Sciences, Tabriz, Iran; 3 Pharmaceutical Analysis Research Center, Tabriz University of Medical Sciences, Tabriz, Iran

**Keywords:** Adulterant, dexamethasone, graphene oxide, herbal supplements

## Abstract

In most cases, herbal supplements found in illegal centers and herbal medicine stores have harmful content that causes irreparable damage to people. As a representative of the green chemistry method, a rapid and efficient dispersive micro solid-phase extraction (D-mSPE) method was applied to extract dexamethasone adulterant from handmade supplements sold as power natural enhancing materials. The quantification of the analyte was performed by high performance liquid chromatography with an ultraviolet detector. Effective parameters on the extraction recovery, including the type and amount of adsorbent, the kind of elution solvent, and the time of extraction and elution, were systematically investigated. The limit of detection of the method was 0.05 μg mL^−1^.

Furthermore, inter-day precisions calculated as relative standard deviations were better than 10%. Ten dietary supplements, including liquid, capsule, tablet forms, and powder, were analyzed using the developed method, and four of them were detected as adulterated with dexamethasone. The developed method purifies the complex plant-based samples and results in a clean analysis with separation instruments.

## 1. Introduction

Over the past few years, dietary supplements, especially those based on herbal and natural ingredients, have become increasingly popular worldwide because they are safer and healthier in the public mind than synthetic supplements. Athletes and interested consumers use supplements to help them lose weight, build muscle and gain immense energy, and companies are trying to tailor their ads to these demands. Dietary supplements are defined as products that supplement the lack or deficiency of one or more essential nutrients in the diet and promote health by improving function or preventing disorders of various organs of the body. Athletes use sports supplements to provide more nutrients, prevent nutrient deficiencies and create energizing effects1] ]. Unfortunately, there is no guideline for approving nutritional supplements and sports supplements. The FDA only approves a supplement or drug if it does not cause severe side effects, and FDA approval does not constitute an effective supplement. Therefore, the responsibility for the purchase lies with the buyer and his level of awareness. Ads about supplements can be misleading. The claim that a supplement is effective may not be reliable.

People take herbal supplements to gain muscle mass, lose weight and improve their performance or general health. They may not be aware that long-term use of supplements can have side effects. Herbal supplements can make some health conditions worse or interfere with medications. Some dietary supplements used in exercise are contaminated. Dexamethasone is a type of corticosteroid. It is used to treat many diseases, including rheumatic problems, skin conditions, severe allergies, asthma, chronic obstructive pulmonary disease, croup, brain swelling, eye pain after eye surgery, and antibiotics in tuberculosis. The physicochemical properties of dexamethasone are provided in Table 1.

Another exciting thing about dexamethasone is that it was illegally added to handmade herbal supplements. For weight gain, corticosteroids, such as dexamethasone pills, are common, especially among young people who go to the gym. Medical experts believe that dexamethasone not only increases muscle mass and strength in athletes but also increases muscle breakdown and weakness. Also, with the use of this drug, the possibility of rupture and damage to the tendons in athletes, during sports movements increases sharply [2]. Many types of supplements that are counterfeit and unlicensed in various perfumeries and stores and are consumed by people without heeding the warnings of the Ministry of Health contain compounds of corticosteroids. These drugs increase the load on kidney function, resulting in irreversible renal and cardiovascular complications and false weight gain, and the athlete thinks that this weight gain is related to muscle building [3]. Perhaps this drug is as popular as an adulterant because, in recent years, dexamethasone has become one of the most common doping drugs in bodybuilding clubs and causes false weight gain. However, this weight gain is due to the accumulation of water and fluids in the body, and it does not affect the increase of muscle mass and athlete strength even its regular consumption leads to a decrease in muscle mass. Since 1996, the use of this drug in championship sports as an energizer and doping drug has been banned [4].

In recent studies, various methods have been introduced to extract dexamethasone from matrices containing it. These methods include immunological methods [6, 5], high-performance liquid chromatography (HPLC) with ultraviolet [8, 7] and fluorescent detectors [9], mass spectrometry [11, 10], and gas chromatography [12] cited. Among the above methods, the HPLC method has been used mainly to extract dexamethasone from biological fluids [13].

The mentioned methods are very suitable for serum and urine analysis. However, few of these methods are ideal for solids and tissue analysis [14]. Due to the complexity of handmade herbal supplements, adulterant analysis in small quantities is one of the significant problems in this field today. Therefore, targeted sample preparation is necessary to detect different types of analytes in these matrices so that the interfering compounds can be removed and preliminary analysis of the analytes performed.

To reduce preparation costs and the consumption of chemical solvents, and to increase the speed and efficiency of sample analysis in quality control laboratories, the solid-phase extraction (SPE) technique leads to more accurate and efficient results than other time-consuming methods. SPE, which is derived from conventional chromatography, used an adsorbent medium to separate the samples based on the difference in equilibrium with the adsorbent medium. In traditional SPE, discs and microfibers are used to extract, which are usually disposable and, in addition to their high cost, are not selective against the target analytes. Therefore, the place of novel extraction techniques along with the analytical instruments is highlighted.

In 2003, Anastassiades et al. first introduced the dispersive micro solid-phase extraction (D-mSPE) method [15]. This method has special advantages over conventional solid-phase extraction (SPE) methods such as an easier extraction process due to no need to pass sample solution or extraction solvent through an SPE column, cost-effectiveness, speed, low organic solvent consumption, and avoidance of column blocking [18–16]. In the D-mSPE method, the adsorbent is mixed directly with the sample through ultrasound, vortex, and other aids. This process leads to a greater contact surface between the adsorbent and the analyte, improving the extraction efficiency. Providing maximum adsorption capacity, minimum cost, and reusability of adsorbents has led to the production of new adsorbents for use in D-mSPE. Using graphene oxide (GO) as an adsorbent has several advantages, such as mechanical and thermal stability, high adsorption capacity, and two-dimensional structures [19]. Oxygen-containing hydrophilic functional groups that contain GO play an important role in modifying GO levels.

One of the limitations of using GO is its very low selectivity. Another major problem in using GO in the extraction process is its agglomeration. The presence of hydrophilic and hydrophobic organic groups either by strong electrostatic repulsive forces or due to their large size prevents the accumulation of GO particles.

Many attempts have been made to covalently and noncovalently functionalize GO [20,21]. However, GO contains several functional groups that can react with different compounds. For example, epoxy groups and carboxyl groups of GO can react with amines, secondary amines, and other compounds containing hydrogen atoms of the hydroxyl group in GO can be replaced by other compounds [22].

In the present work, we report a new pathway for the simultaneous reduction and functionalization of GO using ethylenediamine (NH_2_-CH_2_-CH_2_-NH_2_). This compound can functionalize and reduce GO by a nuclear loop opening reaction. In addition, the reduction and functionalization of GO using ethylenediamine due to the presence of two amine properties (-NH_2_) on both sides of the ethylene portion bind the graphene plates together. This adsorbent is used in D-mSPE process for selective extraction of dexamethasone adulterant from handmade supplements before analysis by HPLC-UV.

## 2. Materials and methods

### 2.1. Materials and solutions

Dexamethasone standard powder was purchased from Scharlau, Spain. Acetonitrile, methanol, acetone, hydrochloric acid, ethylenediamine, ammonia, and sodium hydroxide were purchased from Merck, Germany. Deionized water was provided from Millipore.

To prepare the stock solution, dexamethasone was prepared at a 1000 μg mL^−1^ concentration in methanol. Standard solutions were prepared by further dilution by diluting this solution with methanol. Freshly prepared standard solutions and stock solutions were stored at 4 °C.

### 2.2. Chromatographic condition

HPLC was used to obtain quantitative information and determine the amount of dexamethasone in herbal supplements. A Knauer device connected to a UV detector at 242 nm and a C_18_ column (250 × 4.6 mm, 5μm, Kanuer) with a flow rate of 1 mL min^−1^ was used. The mobile phase consisting of 30% deionized water and 70% acetonitrile was used. The volume of each injection was 25 μL.

### 2.3. Synthesis of adsorbent GO-EDA (graphene oxide-ethylenediamine)

First, GO was synthesized based on the previous work of researchers [23]. Next, 200 mg of graphene oxide is dispersed in 33 mL of water by an ultrasonic device, and then 9 mL of ammonia is added and stirred for 10 min. Then 33 mL of ethylenediamine (containing 600 mg of ethylene diamine dissolved in ethanol) is added and refluxed at 90 ° C for 16 h. The solid-phase was then centrifuged and washed several times with ethanol. Finally, the resulting solid dried in an oven at 60 °C.

### 2.4. Preparation of handmade herbal supplements

Supplemental samples were purchased randomly from herbal medicine stores (Tabriz, Iran). These samples were in the form of powder, beverage solution, tea, or concentrated syrup. In the case of powder samples, all samples were crushed into small pieces. To prepare the powder sample, the bag was opened and the powder was homogenized.

The exact amount of each sample (0.1 g of solid or 1 mL of beverage solution) was prepared. In concentrated syrup-like samples, the sample was diluted 1:1 by volume with water. The samples were transferred to a 2 mL vial, 70% methanol was added, and then placed in an ultrasound device for 5 min. After that, the solid phase was separated in a centrifuge at 5000 rpm for 5 min and 1.5 mL of the supernatant was transferred to the vial for the D-mSPE process.

### 2.5. Dispersive micro solid-phase extraction (D-mSPE) method

The composition of supplements consists of different formulas, such as natural plant extractions. Since herbal matrix contains both lipophilic and hydrophilic compounds, therefore the extraction of adulterants by a selective solvent is impossible. The D-mSPE was applied, and the result of extraction was evaluated in the matter of recovery and matrix effect. Lipid and lipid-like co-existing materials were not bound.

First, 1.5 mL of each sample was placed in a vial. Next, weigh six mg of the adsorbent and add to the vial. The sample vial is placed in the ultrasound device for 4 min. The sample is then centrifuged in a centrifuge for 5 min at 12,000 rpm. The solid adsorbent is removed from the supernatant, and the top solution is discarded. Next, the 150 μL of desorption solvent (acetonitrile) is added to the vial containing the adsorbent. The sample is placed in a sonicator for 5 min and then centrifuged again in a centrifuge at 12,000 rpm. The supernatant is then removed and injected into the HPLC system.

## 3. Results and discussion

### 3.1. Confirmation of GO-EDA structure

**Figure 1:**
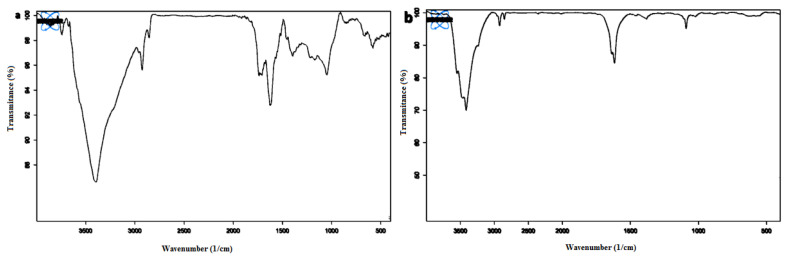
FT-IR analysis of a) GO, and b) GO-EDA.

FT-IR analysis was used to confirm the structure of GO and GO-EDA adsorbents in the range of 500 to 4000 cm^−1^ (Figures 1a and 1b). Figure 1a shows the FT-IR spectrum for bare GO. The broad peak at 3397 cm^−1^ indicates the hydroxyl stretching vibration of the C–OH groups. The FTIR spectrum of GO also has some peaks at 1739 cm^−1^, 1610 cm^−1^, 1400 cm^−1^, and 1032 cm^−1,^ corresponding to the C=O stretching vibrations of the–COOH groups, C=C in the aromatic ring, C–OH group, and C–O–C in the epoxide group, respectively.

In the FT-IR spectrum of GO-EDA (Figure 1b), the absorption peak at 1629 cm^−1^ is related to tensile vibrations of N-H for amid moiety. The broad absorption peak at 3418 cm^−1^ indicates the O-H and N-H stretching bands. The absorption courier in 1022 cm^−1^ corresponds to the C–N (in –C–NH–C– group), which reveals the presence of an amino group on the graphene oxide.

**Figure 2:**
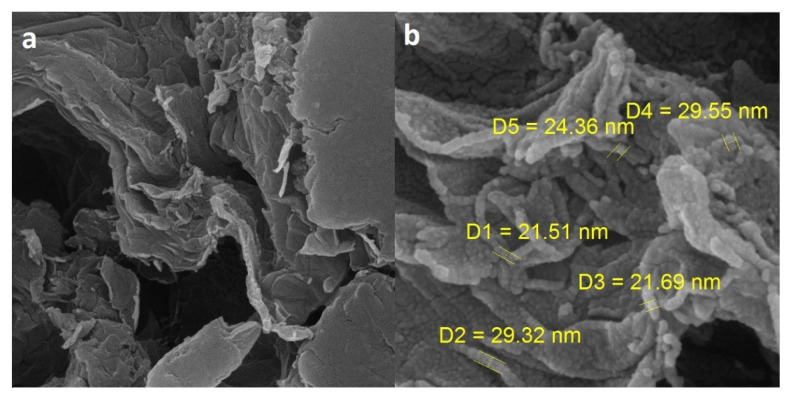
Characterization of the a) GO and b) GO-EDA by scanning electron microscopy (SEM).

The morphology of the adsorbent shows that stacking layers of GO (Figure 2a) after incorporation with EDA form a 3D mseo porous structure (Figure 2b). In addition, the SEM results show that the adsorbent possesses higher surface area and porosity due to the interconnected layers of GO when compared with native GO.

**Figure 3:**
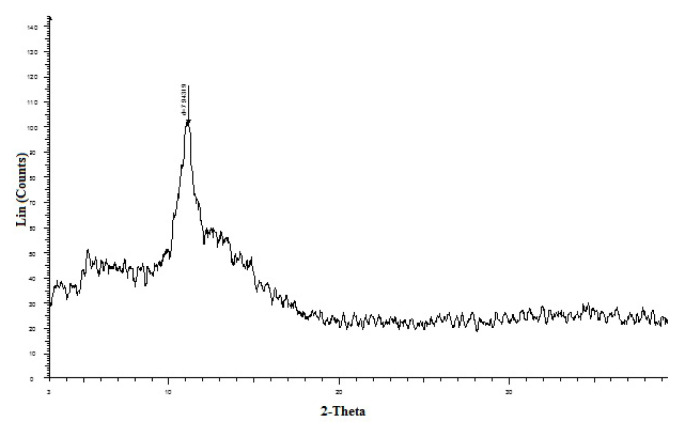
XRD pattern of GO.

In addition, the crystalline properties of the GO were investigated and analyzed using X-ray diffraction (XRD), as shown in Figure 3. The GO shows a typical peak at 2θ = 11.3°, indicating the distance between GO sheets due to functional groups such as hydroxyl, epoxy, and carboxyl groups attached on inter-planner sheets from both sides.

### 3.2. Optimization of effective parameters in dispersive micro solid-phase extraction (D-mSPE) method

To achieve the highest recovery of the D-mSPE method, the most important parameters influencing the extraction process were checked by the one parameter-at-a-time method.

The standard solution is prepared at a concentration of 10 μg mL^−1^ of dexamethasone in a volume of 1.5 mL. The most important parameters included in this study are the type of adsorbent, amount of adsorbent, extraction time and desorption time, and kind of elution solvent. Thus, each of the above parameters has changed within the predetermined range while the other parameters have been kept constant. HPLC-UV records the extraction results, and the area under the curve is recorded for each value. This is done for all parameters, and finally, all the optimized parameters are obtained for extraction.

#### 3.2.1. Adsorbent type

The extraction efficiency of GO-EDA was compared with that of commercially available adsorbents such as C_18_ and bare GO. The results showed that the GO-EDA adsorbent in the analysis by HPLC-UV showed a higher peak area (535,850) than the GO (273,121) and C18 adsorbent (101,019). This is because graphene sheets have a flat hexagonal array of carbon atoms to provide a larger surface area for the interactions between analyte and adsorbent [24]. In addition, EDA groups increase GO plates’ spacing and increase the adsorbent dispersion in the sample matrix due to their hydrophilic properties [25]. Therefore, better distribution of the adsorbent can improve the extraction performance of the adsorbent. The possibility of hydrogen bonding between the analyte and the adsorbent functional groups must also be considered. Silica-based C_18_ adsorbent is more hydrophobic and shows lower extraction efficiency in a polar solvent.

#### 3.2.2. Amount of adsorbent

**Figure 4:**
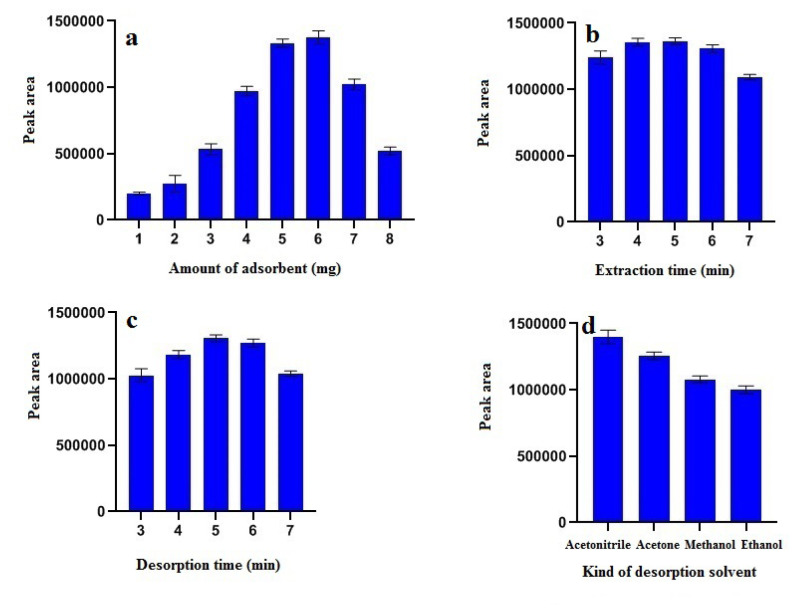
Optimization of the dispersive micro solid-phase extraction (D-mSPE) a) amount of adsorbent, b) extraction time, c) desorption time, and d) kind of desorption solvent.

Optimization of the amount of adsorbent used in the D-mSPE method plays an essential role in improving the efficiency of the extraction method. Nano-sized adsorbents have received more attention than conventional (micro-adsorbents) due to their high surface-to-volume ratio and significant extraction capacity. Therefore, satisfying results can be achieved with smaller amounts of nano-sized adsorbents. To observe the effect of the amount of adsorbent on the signal of the HPLC system, different quantities of GO-EDA adsorbent (from 1 to 8 mg) are added to the dexamethasone standard solution and the peak area related to dexamethasone is calculated. As can be seen in Figure 4a, the extraction efficiency improves with increasing amounts of GO-EDA adsorbent due to the increase in the level and number of active sites. The maximum extraction efficiency of dexamethasone was obtained using 6 mg of GO-EDA adsorbent, and the addition of more than 6 mg of adsorbent did not significantly increase the extraction efficiency. Compared to common adsorbents such as cartridges, disks, etc., used in the solid-phase extraction method, the amount of adsorbent is in milligrams. The provision of the necessary surface for drug extraction is a unique advantage for the D-mSPE method and is considered a diffusion phenomenon. Therefore, in subsequent studies, 6 mg of GO-EDA adsorbent was used as the adsorbent.

#### 3.2.3. Extraction time

In the diffuse solid microextraction process, the extraction time refers to the time between adding the adsorbent to the sample medium until the analyte is fully loaded. The extraction time of the analyte or analytes on the adsorbent determines the adsorption kinetics. The effect of extraction time from 3 to 7 min was investigated. The results in Figure 4b show that by increasing the extraction time from 1 to 4 min, the response of the HPLC system improves and then reaches a constant value. Increasing the extraction time to adsorb the dexamethasone is effective until the adsorption equilibrium is established. Therefore, 4 min was chosen as the extraction time. It should be noted that extraction is a dynamic process and the uptake of dexamethasone may be slightly reduced as the extraction time is prolonged. In 4 min, the extraction reaches equilibrium.

#### 3.2.4. Elution time

The time required to wash the dexamethasone from the adsorbent is another major factor in improving the extraction of dexamethasone. Therefore, the time needed to evaluate the drug desorption was optimized from 3 to 7 min. The effect of desorption time on the amount of desorbed dexamethasone is shown in Figure 4c. Experimental results showed that 5 min was sufficient to wash dexamethasone from the GO-EDA adsorbent.

#### 3.2.5 Type of elution solvent

The type of solvent and adsorption is an essential factor that affects the extraction efficiency [26]. In the solid-phase microextraction process, the recovery of loaded dexamethasone on the adsorbent surface should be investigated. A suitable adsorbent solvent should be able to separate the adsorbed analyte with the least volume without damaging the nature of the adsorbent and analyte surface.

Acetone, methanol, and acetonitrile were desorption solvents used for dexamethasone washing. The results showed (Figure 4d) that acetonitrile is the most suitable desorption solvent in this experiment. Acetonitrile can dissolve dexamethasone more strongly and interact with GO-EDA uptake sites, weakening the interaction between dexamethasone and the adsorbent.

### 3.3. Method validation

To construct the calibration graph, standard dexamethasone solutions were prepared by applying D-mSPE method, and the linear range was plotted at the range of 3.12–50.0 μg mL^−1^. The lower limit of quantification (LLOQ) is defined as the lowest level of the calibration curve, which is 3.12 μg mL^−1^. The limit of detection (LOD) was also calculated at around 0.05 μg mL^−1^. To evaluate the method’s accuracy, three concentrations were selected from the range of the calibration diagram and were repeated three times, the results of which are shown in Table 2. The recovery ranged from 95.3% to 98.0%, and the RSD% values were better than 8%.

### 3.4. Method application

In any new analytical method that is presented, the ability of the method to evaluate and measure analytes in real samples with high accuracy must be considered. Therefore, to assess the reliability of the method, 10 supplements supplied from herbal medicine stores located in Tabriz, Iran were purchased and examined for the presence of dexamethasone.

To ensure that the matrix does not affect the efficiency of the extraction method, the relative recovery percentage was also calculated. The relative recovery percentage was determined by increasing the standard concentration of dexamethasone in the sample matrix. To achieve reliable results, each experiment was performed independently in three replications, and spike samples with a concentration of 5 ppm were also tested.

**Figure 5:**
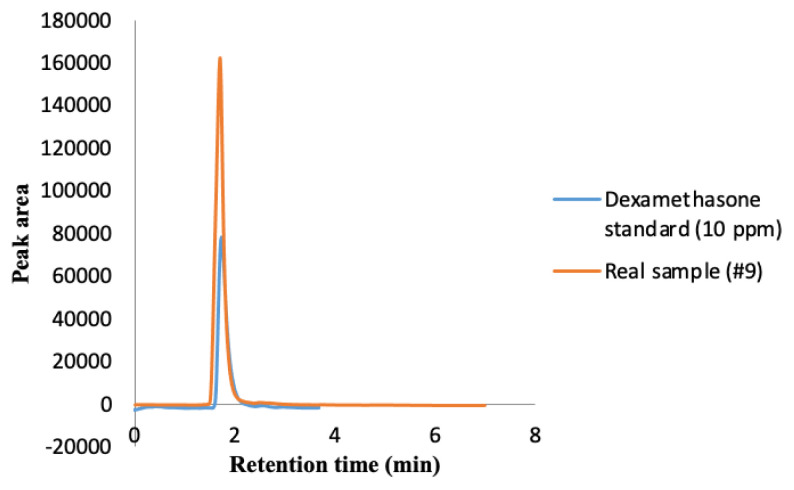
Chromatogram of a real sample (#9) extracted by dispersive micro solid-phase extraction (D-mSPE).

As can be seen in Table 3, RSDs are less than 9%, and recoveries are in the range of 82.7% to 109.4%, indicating that systematic error during the extraction process is very low. Dexamethasone was found in 4 samples in the concentration range of 15.60 to 85.86 μg mL^−1^. The chromatograms of one of the samples where dexamethasone was detected are shown in Figure 5.

### 3.5. Extraction mechanism

The synthesized adsorbent GO-EDA has an aromatic structure that is very stable and does not change quickly. The extraction mechanism mainly involves the hydrophobic interaction between dexamethasone in solution and the adsorbent surface. Dexamethasone is a weak acid; it is protonated in a very acidic environment, which is not in the interest of extraction. It is also very unstable in an alkaline environment. Long-chain fatty acids are the main components in the matrix of supplements, which are mostly plant-based. They have long alkyl chains and are usually present in oligomers larger than 10 nm, while the drug has much smaller dimensions. The adsorbent plates are layered and large molecules are not able to enter. This means that uniform micropores in the adsorbent can effectively block large compounds in the complement matrix and allow relatively small dexamethasone to enter. In addition, the large adsorbent surface may facilitate the uptake of dexamethasone due to the Van der Waals interaction and hydrophobic bonding. The high distribution ratio of water to octanol and the hydrophobic nature of the adsorbent contribute to this. The hydrogen bond between the dexamethasone and the adsorbent surface is also considered to be the driving force of the adsorption process.

### 3.6. Method comparison

To evaluate the developed method objectively and comprehensively, a comparison was made between this method and several other reported methods to determine the amount of dexamethasone. Important influencing factors such as sample preparation method, use of organic solutions, amount of adsorbent used in each test, extraction time, analyzers used, recovery, RSD, LLOQ are presented in Table 4. The results showed that the present method could achieve the desired recovery and reproducibility. Most of the methods in Table 4 require two or more steps to separate by solid-phase extraction, resulting in a tedious, time-consuming organic solvent pretreatment method (usually washing and equilibration steps) [27–29,8]. In contrast, our developed approach can selectively and directly extract dexamethasone from handmade herbal supplementary without any additional steps. Extraction time by the current method was 5 min, shorter or comparable to most reported methods. In addition, disposable absorbents used directly in the cartridge can create additional costs and increase the risk of environmental pollution. The results show that the present method is economical and the amount of adsorbent (adsorbent amount of 6 mg) is less than other solid-phase microextraction methods [30,31] and is also environmentally friendly and only the solvent used is in the range of 100 μL to implement the desorption process. In methods such as kit design that are not based on separation, the possibility of incorrect responses because of cross-reactions should be considered [32]. Examination of the method’s scope designed on complementary handmade compounds showed that the current method shows excellent extraction and purification. The matrix effect is well removed, and LLOQ, recovery, and RSDs eliminate the need for quality control laboratories to control these supplements. Although the current method for dexamethasone extraction is not the official method of QuEChERS, it still shows fast, easy, cheap, effective, and safe properties. The LLOQ of the method is about 3.12 μg mL^−1^, which is improved by coupling the method to a more sensitive detector. This LLOQ is adequate for adutrant investigation in real samples.

## 4. Conclusion

The present study used dispersive micro solid-phase extraction (D-mSPE) based on GO-EDA adsorbent to purify the matrix and concentrate dexamethasone. The adsorption ability of GO-EDA was shown to be higher than that of commercial adsorbents. This simple, fast, and inexpensive method is suitable for determining dexamethasone within the matrix of handmade supplements presented in herbal medicine shops. However, an examination of real samples purchased from herbal medicine shops showed that four out of ten items were contaminated with dexamethasone, indicating the need for more regulatory rules. Goods on the market, including supplements and beverages available at herbal medicine shops, sports supplement centers, and even pharmacies, can contain various illicit substances, whether they are labeled genuine or not.

Previous methods for detecting and measuring dexamethasone can have acceptable efficiencies, but for industry-wide monitoring, other factors arise for us, such as the short time to perform the test. Availability and low cost of required materials, the ability to separate and concentrate the method, and other factors that can make it possible to perform the technique continuously and reliably at the level of laboratories of regulatory bodies. Our proposed method also focuses on these factors using the micro-extraction method. According to studies, this is the first time a solid-phase micro-extraction method with GO-EDA adsorbent is used to extract dexamethasone. This method reduces the number of separation steps and can be considered a fast and green method. The clean extracting is obtained by washing and prevents the matrix from interfering. The results showed acceptable linear range analysis and suitable accuracy for dexamethasone measurements. The present study results will further develop sample preparation methods using carbon-based adsorbents to monitor food quality. The results confirmed the feasibility of the proposed method, which can be easily performed for routine testing and monitoring of supplements offered at the level of perfumers. The product label should be such as to inform the consumer of the controlled items.

## Figures and Tables

**Table 1:** The physicochemical properties of dexamethasone.

Analyte	Chemical structure	Molecular dimension (nm^*^ nm)	Log *k*_ow_	pka	Molecular weight	Chemical formula
Dexamethasone	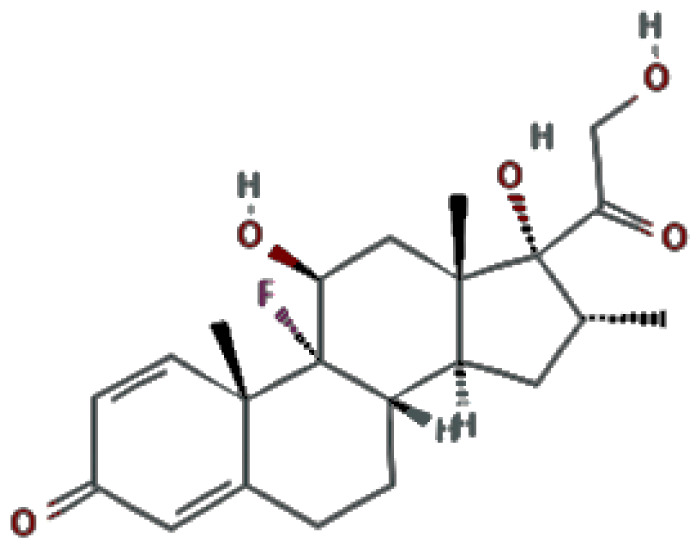	0.85 × 2.00	1.83	12.42	392.5 g/mol	C_22_H_29_FO_5_

**Table 2:** Precision and accuracy of developed dispersive micro solid-phase extraction (D-mSPE).

Dexamethasone concentration μg mL^−1^	Precision (RSD%) (n = 3)	Accuracy (Recovery%)
12.5	7.3	95.3
25.0	1.7	98.0
50.0	1.1	96.5

**Table 3:** Measurements of dexamethasone in real samples by dispersive micro solid-phase extraction (D-mSPE).

Sample #	Spiked dexamethasone (μg mL^−1^)	Determined dexamethasone (μg mL^−1^)	RSD%	Recovery%
1	0	15.60	3.09	–
1	5	4.13	6.19	82.7
2	0	0	–	–
2	5	5.22	4.97	104.45
3	0	0	–	–
3	5	5.03	7.95	100.69
4	0	0	–	–
4	5	4.98	3.79	99.7
5	0	0	–	–
5	5	5.09	4.78	101.9
6	0	34.56	–	–
6	5	4.83	5.81	96.7
7	0	0	–	–
7	5	4.84	2.75	96.88
8	0	0	–	–
8	5	5.47	8.92	109.4
9	0	85.86	1.32	–
9	5	4.307	7.47	86.14
10	0	66.62	7.89	–
10	5	5.34	5.62	106.32
